# Ventilator-associated pneumonia using a heated humidifier or a heat and moisture exchanger: a randomized controlled trial [ISRCTN88724583]

**DOI:** 10.1186/cc5009

**Published:** 2006-08-02

**Authors:** Leonardo Lorente, María Lecuona, Alejandro Jiménez, María L Mora, Antonio Sierra

**Affiliations:** 1 Intensive Care Unit, Hospital Universitario de Canarias, La Laguna, Tenerife, Spain; 2 Department of Microbiology, Hospital Universitario de Canarias, La Laguna, Tenerife, Spain; 3 Research Unit, Hospital Universitario de Canarias, La Laguna, Tenerife, Spain

## Abstract

**Introduction:**

Some guidelines to prevent ventilator-associated pneumonia (VAP) do not establish a recommendation for the preferential use of either heat and moisture exchangers (HMEs) or heated humidifiers (HHs), while other guidelines clearly advocate the use of HMEs. The aim of this study was to determine the incidence of VAP associated with HHs or HMEs.

**Methods:**

A randomized study was conducted in the intensive care unit of a university hospital involving patients expected to require mechanical ventilation for >5 days. Patients were assigned to two groups; one group received HH and the other group received HME. Tracheal aspirate samples were obtained on endotracheal intubation, then twice a week, and finally on extubation, in order to diagnose VAP. Throat swabs were taken on admission to the intensive care unit, then twice a week, and finally at discharge from the intensive care unit in order to classify VAP as primary endogenous, secondary endogenous, or exogenous.

**Results:**

A total of 120 patients were assigned to HMEs (60 patients) and HHs (60 patients); 16 patients received mechanical ventilation for less than five days and were excluded from the analysis. Data analysis of the remaining 104 patients (53 HMEs and 51 HHs) showed no significant differences between groups regarding sex, age, Acute Physiology and Chronic Health Evaluation II score, pre-VAP use of antibiotics, days on mechanical ventilation, and diagnosis group. VAP was found in eight of 51 (15.69%) patients in the HH group and in 21 of 53 (39.62%) patients in the HME group (*P *= 0.006). The median time free of VAP was 20 days (95% confidence interval, 13.34–26.66) for the HH group and was 42 days (95% confidence interval, 35.62–48.37) for the HME group (*P <*0.001). Cox regression analysis showed the HME as a risk factor for VAP (hazard rate, 16.2; 95% confidence interval, 4.54–58.04; *P *< 0.001).

**Conclusion:**

The patients mechanically ventilated for more than five days developed a lower incidence of VAP with a HH than with a HME.

## Introduction

The use of mechanical ventilation with an artificial airway requires conditioning of the inspired gas [1]. This conditioning is necessary because medicinal gases are cold and dry, and when the upper airway is bypassed it cannot contribute to the natural heat and moisture exchange process of inspired gases.

At low levels of inspired humidity, water is removed from mucous and the periciliary fluid by evaporation, causing increased viscosity of mucous and loss of the periciliary fluid layer. Mucociliary clearance therefore decreases since the thick mucous is difficult for cilia to remove; also, mucociliary transport is impaired due to a decreased cilia beat rate. Continuous desiccation of the mucosa causes cilia paralysis, cell damage, and decreased functional residual capacity, and atelectasis may develop.

Artificial humidification of medicinal gases can be active or passive. In active humidifiers, known as heated humidifiers (HHs), the inspired gas passes across or over a heated water bath. Passive humidifiers, known as artificial noses or heat and moisture exchangers (HMEs), trap heat and humidity from the patient's exhaled gas and return some to the patient on the subsequent inhalation.

Some authors advocate an absolute humidity of 26–30 mg water vapour/l gas [2-5] and have recommended the use of HMEs [6-10]. Other authors advocate an absolute humidity of 44 mg water vapour/l and have recommended the use of HHs [11-14]. There is also controversy concerning the possible influence of these systems on the incidence of ventilator-associated pneumonia (VAP). One study reported a lower incidence of VAP associated with the use of HMEs [15]. On the other hand, several studies found no significant differences in the VAP incidence between the two systems [16-25]. There are also previous data with a lower incidence of VAP associated with HHs [26,27]; one of these studies, however, used an obsolete hydrophobic HME [26], and the other is a congress abstract that was never published [27]. Several published guidelines on the prevention of VAP have not established a recommendation for the preferential use of either HMEs or HHs [28-30], while others clearly advocate the use of HMEs [31,32].

The objective of this study was to compare the incidence of VAP using either a HH system or a HME system in patients on mechanical ventilation for more than five days.

## Materials and methods

A randomized study was performed at the 24-bed medicosurgical intensive care unit (ICU) of the Hospital Universitario de Canarias (Tenerife, Spain), a 650-bed tertiary hospital, from 1 January 2005 to 31 December 2005. The study was approved by the Institutional Review Board, and informed consent was obtained from patients or their legal guardians.

We included patients expected to require mechanical ventilation for more than five days. Exclusion criteria were age <18 years, HIV, a blood leukocyte count <1,000 cells/mm^3^, solid or haematological tumour, and immunosuppressive therapy.

Patients were assigned to receive humidification at the time of intubation either with a HME or with a HH, by a random number list generated using Excel software (Microsoft, Seattle, WA, USA). The HME was the Edith Flex^® ^(Datex-Ohmeda, Helsinki, Finland). HME devices were changed at 48-hour intervals. The HHs used were the MR 850^® ^(Fisher&Paykel Health Care Ltd, Auckland, New Zealand) and the Aerodyne 2000^® ^(Tyco Healthcare/Nellcor, Pleasanton, CA, USA), set to deliver a temperature of 37°C and 100% relative humidity to the proximal airway (containing approximately 44 mg water/l gas as per the manufacturer's recommendations). These HHs are servo-controlled humidifiers with wire-heated circuits without water traps and with an autofeed chamber to refill the chamber with water.

In both patient groups identical measures for the prevention of nosocomial pneumonia were established: no routine change of ventilator circuits, a closed tracheal suction system, a semirecumbent body position, continuous enteric nutrition, periodic verification of the residual gastric volume, prophylactic ranitidine for stress ulcers, oral washing with clorhexidine, no selective digestive decontamination, and no aspiration of subglottic secretions.

Tracheal aspirate samples for analysis were obtained on endotracheal intubation, then twice a week, and finally on extubation, in order to diagnose VAP. Throat swabs were taken on admission to the ICU, then twice a week, and finally at discharge from the unit in order to classify VAP as primary endogenous, secondary endogenous, or exogenous. Necessary clinical samples were taken, entirely independent of how many clinical samples were processed for microbiological analysis.

The diagnosis of pneumonia was established when all of the following criteria were fulfilled: new onset of bronchial purulent sputum, a body temperature >38°C or <35.5°C, a white blood cell count >10,000 mm^3 ^or <4,000/mm^3^, a chest radiograph showing new or progressive infiltrates, and a significant culture of respiratory secretions by tracheal aspirate (>10^6 ^cfu/ml).

Pneumonia was considered as VAP when it was diagnosed after 48 hours of mechanical ventilation. VAP was considered as primary endogenous when caused by microorganisms already present in the patient's oropharyngeal flora on admission to the ICU. VAP was considered as secondary endogenous when caused by microorganisms not found on admission but detected in the patient's oropharyngeal flora during the ICU stay. VAP was considered as exogenous when it was caused by microorganisms that were never carried in the patient's oropharyngeal flora.

The following variables were taken from each patient: sex, age, diagnosis group, Acute Physiology and Chronic Health Evaluation II score, duration of mechanical ventilation, antibiotics previous to VAP, transport out of the ICU, paralytic agents, tracheostomy, reintubation, and mortality.

We found in a previous study [33] that the proportion of patients developing pneumonia after >5 days of mechanical ventilation was 36% using HMEs. For a power of 80% and a 5% type I error, we needed 40 patients per group to test the proportion of patients needed to reduce this proportion (36% using HMEs) to 12% using HHs. We assumed a drop-out rate of 33% (patients <5 days of mechanical ventilation) per group. With this condition, we needed to include 60 patients per group.

Quantitative variables are reported as the mean ± standard deviation, and were compared with the Student *t *test. Qualitative variables are reported as percentages, and were compared with the chi-squared test or with Fisher's exact test as appropriate. The probability of remaining free of VAP was calculated using the Kaplan-Meier method, and comparison between the two groups was performed with the log-rank test. Five Cox proportional hazard models were constructed for the following dependent variables: VAP-free time, VAP by Gram-positive cocci-free time, VAP by Gram-negative bacilli-free time, endogenous primary VAP-free time, and endogenous secondary VAP-free time. The effect of the Acute Physiology and Chronic Health Evaluation II score was controlled in the five models because the *P *value was less than 0.20 in the univariate analysis. The main independent variable in the five models was the type of humidification system (HH versus HME). The significant variables were selected using a forward conditional method. *P *< 0.05 was considered statistically significant. For statistical analyses we used SPSS 12.0.1 software (SPSS Inc., Chicago, IL, USA) and StatXact 5.0.3 software (Cyrus Mehta and Nitin Patel, Cambridge, MA, USA).

## Results

A total of 120 patients were randomly assigned to receive HME (*n *= 60) or to receive HH (*n *= 60). Of these 120 patients, 16 patients were excluded from the analysis because they received mechanical ventilation for less than five days: in the HME group, six patients were extubated earlier and one patient died; and in the HH group, eight patients were extubated earlier and one patient died.

**Figure 1:**
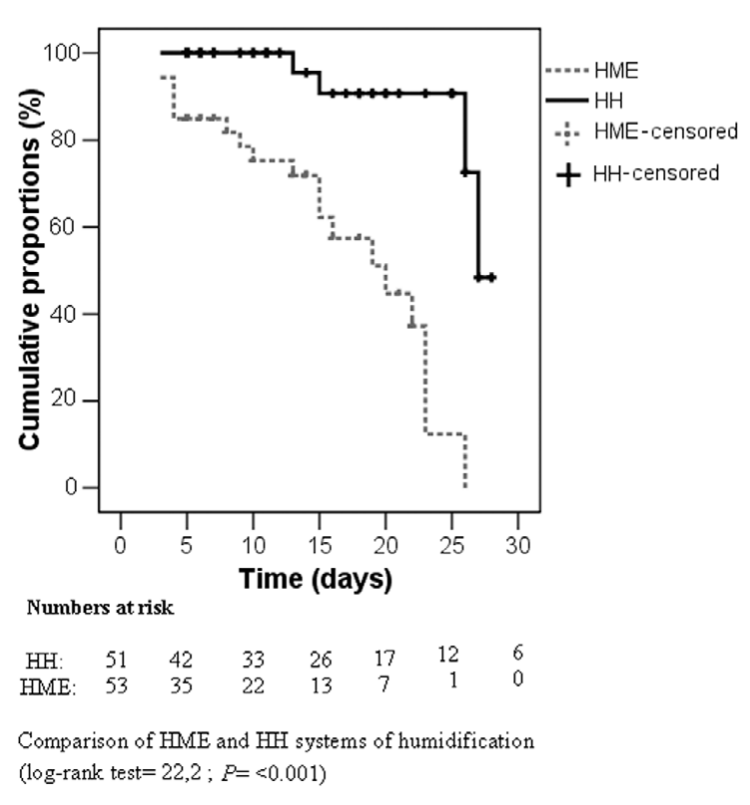
Cumulative proportion of patients remaining free of ventilator-associated pneumonia using heat and moisture exchangers (HMEs) versus heated humidifiers (HHs).

A total of 104 patients received mechanical ventilation for more than five days (53 patients with HMEs and 51 patients with HHs) and were analyzed. There were no significant differences between groups with respect to sex, age, Acute Physiology and Chronic Health Evaluation II score, use of antibiotics prior to VAP, days on mechanical ventilation, and diagnosis group (Table 1). VAP occurred in eight of 51 (15.69%) patients in the HH group and in 21 of 53 (39.62%) in the HME group (*P *= 0.006). Kaplan-Meier analysis confirmed a significantly lower incidence of VAP in the HH group than in the HME group (log-rank test = 22.2, *P *< 0.001) (Figure 1). The median time free of VAP was 20 days (95% confidence interval, 13.34–26.66) for the HH group and was 42 days (95% confidence interval, 35.62–48.37) for the HME group (*P <*0.001). The multivariate Cox regression analysis showed the HME as a risk factor for VAP (hazard rate, 16.2; 95% confidence interval, 4.54–58.04; *P *< 0.001), for VAP caused by Gram-positive cocci, for VAP caused by Gram-negative bacilli, for primary endogenous VAP and for secondary endogenous VAP (Table 2).

Table 3 presents the microorganisms responsible for VAP (18 Gram-negative bacilli and 11 Gram-positive cocci) and the pathogenesis of VAP according to oropharyngeal flora (nine primary endogenous, 18 secondary endogenous and two exogenous). In the HME group we found 13 patients with VAP caused by Gram-negative bacilli and eight patients with VAP caused by Gram-positive cocci; the pathogenesis of VAP according to oropharyngeal flora was primary endogenous in eight cases, secondary endogenous in 12 cases, and exogenous in one case. In the HH group we found five patients with VAP caused by Gram-negative bacilli and three patients with VAP caused by Gram-positive cocci; the pathogenesis of VAP according to oropharyngeal flora was primary endogenous in one case, secondary endogenous in six cases, and exogenous in one case.

## Discussion

There are two meta-analyses suggesting an association between the use of HME and a decreased VAP rate [34,35], although only the study of Kirton and colleagues [15] reported a significantly lower incidence of VAP with HMEs compared with HHs. These authors indicate that this effect may be attributed to two mechanisms [15]: the inclusion of a specifically designed microbiological gas filter in the HME, which it is suggested protects the patient from exogenous VAP; and reduced contaminated condensate in the HME circuit [16,19].

In relation to the first mechanism, we think that convincing outcome data are currently insufficient to support the role of gas filtration in reducing the incidence of VAP [36-38]. Previous studies evaluating the effect of gas filtration in anaesthesia machines [36,37] and in ventilators [38] were unable to demonstrate differences in the incidence of VAP between the patient groups with and without filters. In relation to the second mechanism, we agree that the entry of the contaminated condensate circuit into the airway may explain the higher incidence of VAP reported with the HH system. For this reason, in the present study a servo-controlled humidifier was used, which differs from a cascade humidifier in that it has a dual-heated circuit (so the mobile circuit condensate is minimal) and it has an autofeed chamber (eliminating the need to open the circuit to refill the chamber with water), which minimizes the possibility of exogenous microorganisms entering the circuit and causing exogenous VAP.

The reduction of VAP found in our study when using HHs as compared with HMEs may be attributed to three causes. The previously mentioned improvement of the HH system (with a dual-heated circuit and an autofeed chamber) is one such cause. Secondly, the present study analyzed patients on mechanical ventilation for more than five days, and the mean duration of mechanical ventilation (20 days) was higher than in previous studies (4–14 days). Finally, with HHs it is possible to deliver higher levels of humidity to the airway (44 mg water vapour/l gas), and several authors believed that these levels can facilitate maximal mucociliary clearance [14-18].

The results of several studies suggest that humidification is preferable with HHs, reporting a lower incidence of tube occlusion [16,17,26,39], a lower incidence of thick bronchial secretions [16,18,40] and a lower incidence of atelectasis [26] than patients with HMEs. In the study by Hurni and colleagues [41], the state of the ciliated bronchial epithelium, obtained by endotracheal aspirate, was scored on the first day and on day five of mechanical ventilation. In both the HH and the HME groups, patients' scores significantly decreased from day one to day five, and the authors noted that they could not exclude some contribution of an inadequate tracheal temperature and inadequate humidity to this progressive reduction in the cytological score. Also, the reduction of the epithelium score was greater, although not significantly so, in the patient group with the HME system than in the patient group with the HH system. We think that the absence of a significant difference may be for two reasons: the sample size was only 41 patients, and the inspired gas in the HH group was conditioned to a relative humidity of 100% and a temperature of only 32°C (what would the result be with a temperature of 37°C that ensures the delivery of approximately 44 mg water/l gas?).

The lower incidence of VAP found in our study when using HHs as compared with HMEs, both primary endogenous VAP (mostly early onset) and secondary endogenous VAP (mostly late onset), may be attributed to the fact that a HME does not maintain optimal humidification and mucociliary transport beyond 24–48 hours of mechanical ventilation. The review by Williams and colleagues [1], which considered 200 articles/texts on respiratory tract physiology and humidification, reveals that there are few humidity, temperature, and mucosal function studies of human subjects and that the duration in most of them was only 12 hours. The authors proposed that the optimal temperature was that of the body and 100% relative humidity. Exposure times longer than 24 hours need to be studied to fully verify this proposition. The trend in the data of absolute humidity versus exposure time map produced from published series leads us to believe that mucociliary dysfunction can occur later than after 24–48 hours with an absolute humidity level <32 mg/l (the humidification delivered by HMEs).

The present study has several limitations. First, we did not perform a direct assessment of gas heating and humidification in the patient, so the airway temperature and humidity were not monitored (we assumed the reliability of the data reported by the manufacturers). Second, we did not perform indirect assessment of gas heating and humidification in the patient, so we did not examine secretion characteristics or possible epithelial bronchial damage. Finally, the VAP diagnostic procedure was not invasive and we used only tracheal aspirate samples.

## Conclusion

Patients mechanically ventilated for more than five days developed a lower incidence of VAP with a HH than with a HME.

## Key message

• Patients mechanically ventilated for more than five days developed a lower incidence of VAP with a HH than with a HME.

## Abbreivation

HH = heated humidifier; HME = heat and moisture exchanger; ICU = intensive care unit; VAP = ventilator-associated pneumonia.

## Competing interests

The authors declare that they have no competing interests.

## Authors' contributions

LL was responsible for the conception and design of the study, for data collection, and for analysis and interpretation of the results. ML was responsible for the conception and design of the study, data collection and interpretation of the results. AJ performed statistical analysis and interpretation of the results. MLM and AS were responsible for the conception and design of the study and the interpretation of the results. All authors approved the final version of the manuscript to be published

## Figures and Tables

**Table 1:** Characteristics of the heat and moisture exchanger and heated humidifier groups of patients

	Heat and moisture exchanger group (*n *= 53)	Heated humidifier group (*n *= 51)	*P *value
Sex, female	21 (39%)	19 (37%)	0.84
Age (years)	55.47 ± 19.83	55.94 ± 20.24	0.90
Diagnostic group			
Cardiology	13 (24%)	11 (21%)	0.82
Respiratory	13 (24%)	16 (31%)	0.51
Neurologic	11 (20%)	12 (23%)	0.81
Trauma	16 (30%)	12 (23%)	0.51
Acute Physiology and Chronic Health Evaluation II score	18.11 ± 2.43	18.72 ± 2.33	0.19
Duration of mechanical ventilation (days)	19.47 ± 16.44	20.82 ± 17.05	0.68
Antibiotics previous to ventilator-associated pneumonia	35 (66%)	34 (66%)	0.99
Transport out of the intensive care unit	28 (52%)	29 (56%)	0.41
Paralytic agents	12 (22%)	12 (23%)	0.55
Tracheostomy	15 (28%)	16 (31%)	0.45
Reintubation	8 (15%)	8 (15%)	0.57
Exitus	13 (24%)	12 (23%)	0.99

Data presented as *n *(%) or mean ± standard deviation.

**Table 2:** Risk of ventilator-associated pneumonia (VAP) for heat and moisture exchangers versus heated humidifiers, adjusted for the Acute Physiology and Chronic Health Evaluation II score

	Heat and moisture exchanger group (*n *= 53)	Heated humidifier group (*n *= 51)	Hazard ratio (95% confidence interval)	*P *value
Global VAP	21 (39%)	8 (15%)	16.20 (4.54–58.04)	<0.001
VAP caused by Gram-positive cocci	8 (15%)	3 (5%)	5.44 (1.08–27.31)	0.04
VAP caused by Gram-negative bacilli	13 (24%)	5 (9%)	23.54 (2.98–186.07)	0.003
Primary endogenous VAP	8 (15%)	1 (1%)	8.56 (1.07–68.70)	0.04
Secondary endogenous VAP	12 (22%)	6 (11%)	12.45 (2.65–58.38)	0.001

Data presented as *n *(%). *P *values from the Cox Regression model.

**Table 3:** Microorganisms isolated in ventilator-associated pneumonia

Microorganism	Heat and moisture exchangers	Heated humidifiers
Total Gram-positive cocci	8 (8 primary endogenous)	3 (1 primary endogenous and 2 secondary endogenous)
Methicillin-sensitive *Staphylococcus aureus*	5 (5 primary endogenous)	1 (1 primary endogenous)
Methicillin-resistant *Staphylococcus aureus*	0	1 (1 secondary endogenous)
*Streptococcus pneumoniae*	3 (3 primary endogenous)	0
*Streptococcus faecalis*	0	1 (1 secondary endogenous)
Total Gram-negative bacilli	13 (12 secondary endogenous and 1 exogenous)	5 (4 secondary endogenous and 1 exogenous)
*Pseudomonas aeruginosa*	5 (5 secondary endogenous)	2 (1 secondary endogenous and 1 exogenous)
*Escherichia coli*	0	1 (1 secondary endogenous)
*Klebsiella *spp.	3 (2 secondary endogenous and 1 exogenous)	0
*Enterobacter *spp.	2 (2 secondary endogenous)	1 (1 secondary endogenous)
*Serratia marcescens*	3 (3 secondary endogenous)	0
*Proteus mirabilis*	0	1 (1 secondary endogenous)
Total	21 (8 primary endogenous, 12 secondary endogenous and 1 exogenous)	8 (1 primary endogenous, 6 secondary endogenous and 1 exogenous)
